# The Whiplash Disease Reconsidered

**DOI:** 10.3389/fneur.2022.821097

**Published:** 2022-03-10

**Authors:** Jens Astrup, Finn Gyntelberg

**Affiliations:** Department of Neurology, Bispebjerg Hospital, University of Copenhagen, Copenhagen, Denmark

**Keywords:** whiplash, spinal dyssynergia, EMG, spinal muscles, neck pain, headache, muscular tenderness

## Abstract

The natural course of the whiplash disease is reconsidered in relation to the predominant view of its cause. It is assumed that a whiplash-type trauma is causing an acute tissue injury such as a distortion or sprain in the neck followed by neck pain and headache, which then tends to become a chronic pain condition. We conclude that the whiplash disease typically evolves following a minor trauma without any signs of a tissue injury. It presents with central neuromotor dysfunction, such as electromyography (EMG) hyperactivity and abnormal activation patterns associated with dyscoordination of the involved and adjacent muscle groups. This indicates a central neurological rather than a peripheral traumatic pathology. This view places the cause of the whiplash disease within the central nervous system, and, in concordance with the EMG abnormalities and motor dyscoordination, we suggest the term cervical spinal dyssynergia for this pathology. It provides a new paradigm for further investigations of this disease as well as a window for possible specific neuropharmacological therapy directed towards dysfunctional neuromotor control.

## Introduction

The whiplash disease is commonly regarded as a posttraumatic condition initiated by distortion and sprain in the neck by a whiplash-type trauma. This tissue injury is supposed to be the cause of the acute symptoms, which then may be followed by secondary neuromodulation of the central nociceptive perception, causing a chronic condition of central hypersensibilisation resulting in lowered pain threshold and hyperalgesia (1). However, verification of the assumed initial tissue injury in the neck appears elusive as evidenced by the accumulating number of negative MRI studies. Also, details about the initial incident, the initial clinical presentation, and later chronicity indicate a disease mechanism other than a traumatic tissue lesion. It, therefore, seems reasonable to reconsider the natural history of the whiplash disease and propose a possible alternative disease mechanism. This reconsideration is based on evidence selected to focus on specific characteristics of the whiplash disease, and not on a systematic review of the whiplash literature.

## The Natural Course of the Whiplash Disease

### Negative MRI Studies

Several MRI studies of the neck have failed to show any traumatic or other specific changes both in the acute and the chronic states. A study by Kongsted et al. (2) of 178 patients in the acute phase and after 3 months concluded that “trauma-related MRI findings are rare.” Earlier studies indicated that whiplash trauma may damage the alar ligaments (3), as well as the tectorial and atlanto-occipital membranes (4). However, these studies had a rather low inter-observer agreement, and, when repeated with blinded radiologists in groups of neck pain with and without whiplash and controls without neck pain, no specific differences could be observed (5, 6). These negative results argue against any initial traumatic tissue injury.

### Lack of Correlation Between Impact Energy and Clinical Symptoms

A study by Castro et al. (7) indicated that 60% of whiplash cases occur at speeds <15 km/h and appear more frequently when only a relatively slight vehicle deformation occurs. In fact, most whiplash cases occur at low speed and low impact energy collisions with either weak or no “dose-response” between impact severity and outcome. Radanov et al. (8) in their detailed analysis of the impact and initial clinical presentation in 117 patients reported that impact severity was categorised as “trivial” in 44% and was not predictive of late recovery. These observations argue against an initial sprain lesion, which, expectedly, would bear some correlation with trauma energy.

### Initial Symptom Delay

The initial clinical presentation includes delays as reported by Radanov et al. (8) in the development of headache and neck pain. They reported delays in the onset of the headache of 10 h and in neck pain of 11 h after the impact in the patients who recovered within 2 years, and delays of 4 and 8 h, respectively, in the non-recovery patients, the variation ranging from minutes to days. These symptom delays favour a slowly evolving pathology rather than an immediate sprain lesion.

### Lack of Specific Therapy

It has been rather disappointing that various rehabilitation programmes in the initial phase of whiplash appear ineffective and, in fact, tend to slow recovery. This applies to physical therapy and multidisciplinary in- or outpatient rehabilitation programmes (9) and other patterns of care provided by GP's, chiropractors, and other specialists (10). There was even a trend in these studies that the more intensive the therapy, the more it slows down recovery. A sprain lesion, however, would expectedly make a good initial recovery enhanced by rest and physical therapy.

### A High Percentage of Chronicity

Chronicity is very high in whiplash compared to other acute sprain lesions elsewhere in the body, such as distortions or sprain of muscle fibres in the extremities. Radanov et al. (8) followed 117 consecutive cases of acute whiplash and observed that 24% were still symptomatic after 1 year and 18% after 2 years. However, chronicity maybe even higher. A study by Kasch et al. (11) indicates that the risk of symptoms after 1 year is about 40% in “high risk” and about 15% in “low risk” groups. The Bone and Joint Decade 2000–2010 Task Force on Neck Pain (12) indicates a similarly high percentage of chronicity in whiplash after 1 year or longer based on a review of 226 articles related to prognostic factors. A study of 446 whiplash Grade 2 patients from Ontario, Canada, free of litigation and from seeking compensation and followed for 2 years, showed chronicity to be about 40% (13).

### A Disease Mechanism Alternative to Trauma?

The natural course of the whiplash disease such as indicated above is discordant with the view of a primary traumatic tissue injury caused by the initial incidence. In particular, the initial delay of symptoms is indicative of a developing rather than an instantly appearing pathology. Consequently, an alternative disease mechanism should be considered. It is alternative in the sense that it should explain an evolving pain process in absence of an acute tissue injury as well as take the EMG abnormalities and the dysfunctional neuromotor control of the involved and adjacent muscle groups into consideration. We suggest an evolving neurological disorder such as spinal cervical dyssynergia as a possible alternative pathology. A dyssynergia in this context refers to a dysfunctional but not dystonic neuromotor control, leading to muscular tenderness and pain, and dyscoordination of movements. Arguments in favour of this alternative pathology come from EMG and head, hand, and eye movement studies.

### Neck Muscle EMG and Activation Pattern in Whiplash

EMG recorded by surface electrodes has been applied in patients with whiplash during various schemes of voluntary head movements. The overall impression is that the EMG activity as indicated by the root mean square amplitude is increased in patients with whiplash (14–19), and that this increase is already present after 1 month of the injury and (14, 15). An altered activation pattern was demonstrated as increased activation of synergistic and accessory muscles (18, 19). The cervical motor dysfunction in whiplash in terms of reduced mobility, disturbed kinesthesia, and altered EMG activity was reviewed by Daenen et al. (20) and by Hesby et al. (21).

### Dyscoordination of Neck Muscles and Adjacent Muscle Groups

Several studies have observed subclinical but significant dyscoordination of head movements in whiplash as measured by “the fly method” (22, 23) and recently by laser tracking (24). Other muscular systems adjacent to the neck musculature are also affected. This is indicated by dyscoordination of eye movements (25, 26) and the upper extremity (24, 27, 28). The dyscoordination of head movements probably is indicative of neuromotor hyperactivity and altered activation patterns as observed in the EMG studies.

## Discussion and Conclusions

The main result of this reconsideration of the whiplash disease is that it is not a posttraumatic disorder caused by an initial traumatic cervical distortion or sprain injury in the neck. Signs of tissue injury are absent in the acute phase. Instead, the natural history of the disease indicates an evolving pathology over hours to days after the initial incident, leading to muscular pain and tenderness and dysfunctional neuromotor control of neck muscles as well as adjacent muscle groups. This seems better explained as an evolving central neurological disorder, and, in concordance with the EMG abnormalities and motor dyscoordination, we suggest the term cervical spinal dyssynergia for this pathology.

This consideration raises the question of how a trivial injury can initiate a dysfunction in the central nervous system. Radanow et al. (8) observed that, in 78% of the patients, the initial trauma occurred without forewarning, suggesting an element of fright in contrast to traumas of similar energy when riding bumping cars in an amusement park. So, an emotional experience of fear and fright may be a prerequisite. The same may also apply to the lumbar region. Low back pain may develop following low-velocity motor accidents similar to a whiplash-type accident (29), indicating that this type of lesion proposed about the whiplash disease is not specific for the cervical region.

The presented reconsideration of the whiplash disease assumes that the muscular tenderness develops second to the dysfunctional neuromuscular innervation, including both hyperactivity and abnormal activation patterns. In analogy, muscular tenderness is significant in patients with cervical dystonia (spasmodic torticollis) (30), presenting with an abnormal dystonic activation pattern. In these patients, functional MRI of the brain reveals functional abnormalities (31). Similar studies in patients with whiplash are warranted. Hopefully, the present perspective of a central nervous system disorder being the causative pathology of the whiplash disease may provide a new paradigm for further investigations of this disease. Also, it may open a window for specific neuropharmacological interventions directed towards the dysfunctional neuromotor control.

## Data Availability Statement

The original contributions presented in the study are included in the article/supplementary material, further inquiries can be directed to the corresponding author/s.

## Author Contributions

JA has prepared the manuscript. FG has assisted in preparing the manuscript. All authors contributed to the article and approved the submitted version.

## Conflict of Interest

The authors declare that the research was conducted in the absence of any commercial or financial relationships that could be construed as a potential conflict of interest.

## Publisher's Note

All claims expressed in this article are solely those of the authors and do not necessarily represent those of their affiliated organizations, or those of the publisher, the editors and the reviewers. Any product that may be evaluated in this article, or claim that may be made by its manufacturer, is not guaranteed or endorsed by the publisher.
